# Circadian regulation of diel vertical migration (DVM) and metabolism in Antarctic krill *Euphausia superba*

**DOI:** 10.1038/s41598-020-73823-5

**Published:** 2020-10-08

**Authors:** Fabio Piccolin, Lisa Pitzschler, Alberto Biscontin, So Kawaguchi, Bettina Meyer

**Affiliations:** 1 Alfred Wegener Institute Helmholtz Centre for Polar and Marine Research, Section Polar Biological Oceanography, Am Handelshafen 12, 27570 Bremerhaven, Germany; 2 Department of Biology, University of Padova, Via Ugo Bassi 58/b, 35121 Padova, Italy; 3 Australian Antarctic Division, Department of the Environment and Energy, 203 Channel Hwy, Kingston, TAS 7050 Australia; 4 Institute for Chemistry and Biology of the Marine Environment, University of Oldenburg, Carl-von-Ossietzky-Strasse 9-11, 26111 Oldenburg, Germany; 5 Helmholtz Institute for Functional Marine Biodiversity at the University of Oldenburg, Ammerländer Heerstrasse 231, 26129 Oldenburg, Germany

**Keywords:** Ecology, Physiology, Zoology, Ecology, Ocean sciences

## Abstract

Antarctic krill (*Euphausia superba*) are high latitude pelagic organisms which play a key ecological role in the ecosystem of the Southern Ocean. To synchronize their daily and seasonal life-traits with their highly rhythmic environment, krill rely on the implementation of rhythmic strategies which might be regulated by a circadian clock. A recent analysis of krill circadian transcriptome revealed that their clock might be characterized by an endogenous free-running period of about 12–15 h. Using krill exposed to simulated light/dark cycles (LD) and constant darkness (DD), we investigated the circadian regulation of krill diel vertical migration (DVM) and oxygen consumption, together with daily patterns of clock gene expression in brain and eyestalk tissue. In LD, we found clear 24 h rhythms of DVM and oxygen consumption, suggesting a synchronization with photoperiod. In DD, the DVM rhythm shifted to a 12 h period, while the peak of oxygen consumption displayed a temporal advance during the subjective light phase. This suggested that in free-running conditions the periodicity of these clock-regulated output functions might reflect the shortening of the endogenous period observed at the transcriptional level. Moreover, differences in the expression patterns of clock gene in brain and eyestalk, in LD and DD, suggested the presence in krill of a multiple oscillator system. Evidence of short periodicities in krill behavior and physiology further supports the hypothesis that a short endogenous period might represent a circadian adaption to cope with extreme seasonal photoperiodic variability at high latitude.

## Introduction

Life on Earth is marked by several environmental rhythms and cycles, like the day/night cycle and the seasonal cycle. Most living organisms have evolved endogenous (i.e. internal) clocks that optimize the synchronization of physiology and behavior with the rhythmic changes in their habitat^1^. The best-known endogenous clock is the circadian clock, which regulates biological rhythms on a daily level^2^. Most of our knowledge about the circadian clock comes from studies on terrestrial model organisms like the fruit fly and the mouse and little is known about circadian regulation in marine organisms^3^. In the marine environment, additional environmental cycles occur, like the tidal cycle which is related to the phases of the moon, and increasing evidence indicates that endogenous tidal and lunar clocks might be present^4,5^. Very little is known about endogenous clocks in high-latitude pelagic organisms, which are exposed to extreme seasonal variations in daylength, food availability and sea-ice cover.

Antarctic krill, *Euphausia superba*, (hereafter krill) are a key zooplankton species in the high-latitude ecosystem of the Southern Ocean due to their pan-Antarctic distribution and their exceptional abundance (the total biomass is estimated to be around 379 Mt)^6^. They play a central role in the Antarctic food web, especially within the southwest Atlantic sector and in the West Antarctic Peninsula region, where up to 70% of the biomass is located^7^. The adaptive success of krill strongly relies on their ability to develop rhythmic biological functions which allow for advantageous temporal synchronization between life-history traits and local environmental cycles^8–10^.

Among these rhythmic functions, diel vertical migration (DVM) plays a major role at the daily level^6,11^. During DVM, krill migrate towards the surface at night to graze on phyto- and zooplankton, respectively and return towards the deeper layers during the day to reduce the risk of being spotted by visually-hunting predators^12^. DVM is not only a krill behavior, but it is widespread within most aquatic ecosystems around the world and has a profound impact on marine communities and marine biogeochemical cycles^13,14^. Krill also display daily cycles of metabolic activity and transcription, which are supposed to maximize the benefits of DVM by enhancing energy-related processes during the night^15–17^.

Previous studies suggested that krill daily rhythms might be under the control of an endogenous circadian clock, as already proposed for other high-latitude zooplankton species^16–19^. During the last years, increasing molecular resources allowed the dissection of the circadian clockwork of krill. A recent analysis of krill circadian transcriptome revealed that krill’s clock might be characterized by an endogenous free-running oscillation with a period of about 12–15 h^20–22^. In terrestrial model organisms, the endogenous free-running oscillation of the clock usually displays a period of about 24 h and it is unclear why the clock in krill should display a shorter endogenous period^2^. One emerging hypothesis is that this might represent a circadian adaption for krill living at high latitudes and might help the clock to entrain to a wide range of photoperiods, for example during summer and winter, when light conditions in the Southern Ocean become extreme^16,22^. However, the exact mechanism by which this should occur is still unknown. In particular, it remains unclear if and to what extent putative clock-regulated output functions in krill (e.g. DVM and oxygen consumption) might be influenced by the shortened endogenous period in the clock.

In this study, we monitored daily rhythms of vertical migration, oxygen consumption and gene expression of the main clock components in krill exposed to simulated natural light/dark cycles (LD) and to constant darkness (DD) to estimate the endogenous period.

## Results and discussion

### Vertical migration

**Figure 1:**
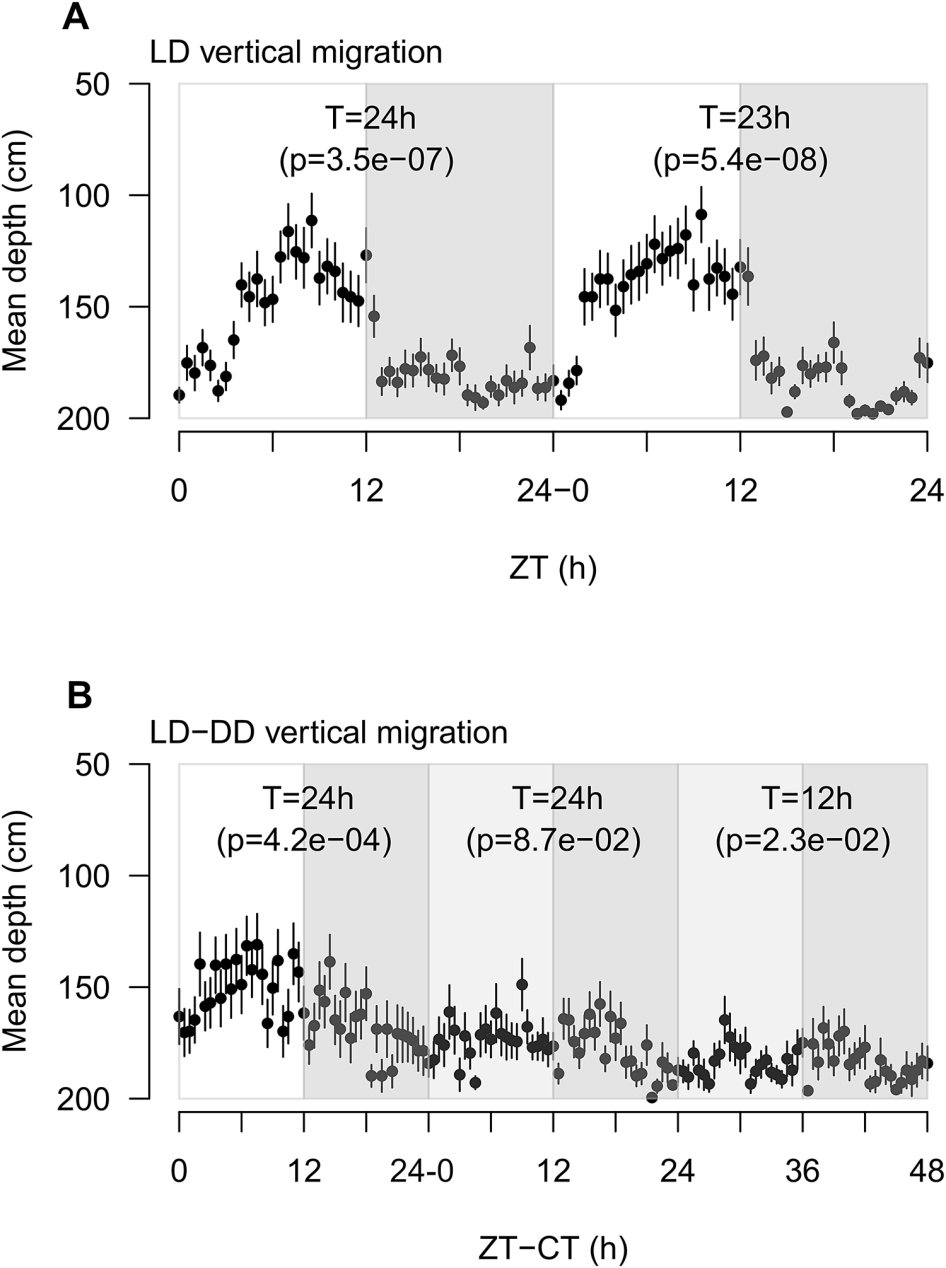
Vertical migration patterns of krill exposed to LD and LD-DD. (**A**) Vertical migration patterns of krill exposed to LD for 48 h. X-axis indicate the Zeitgeber Time (ZT) given in hours from each event of lights-on (6:00), corresponding to ZT0. Y-axis indicate the mean krill depth in cm (n = 45). Error bars represent SEMs. White and grey rectangles represent alternation of light and dark phases. For each experimental day, results of the RAIN analysis test for the presence of rhythmic oscillations are reported (T = period of oscillation; p = p-value). (**B**) Vertical migration patterns of krill exposed to LD for 24 h followed by 48 h in DD. X-axis represent the time in hours from the beginning of the run. For the first 24 h, time is given in ZT, with ZT0 corresponding to lights-on (6:00). For the following 48 h, time is given in Circadian Time or CT, with CT0 corresponding to subjective lights-on in DD (6:00). Y-axis indicate the mean krill depth in cm (n = 41). Error bars represent SEMs. White, grey and light grey rectangles represent alternation of light, dark and subjective light phases respectively. For each experimental day, results of the RAIN analysis test for the presence of rhythmic oscillations are reported (T = period of oscillation; p = p-value). The figure was generated using the *plot* function in the “graphics” package (version 3.6.3, https://www.rdocumentation.org/packages/graphics) in R (RStudio version 1.0.136, RStudio Team 2016).

We performed two separate vertical migration experiments (Fig. 1A,B). In the first one (Fig. 1A) a group of 45 krill was exposed to LD for 48 h. In the second one (Fig. 1B) a group of 41 krill was exposed to LD for 24 h followed by constant darkness (DD) for 48 h. During LD, lights were turned on at 6:00 (corresponding to Zeitgeber Time 0 or ZT0). Light intensity gradually increased until 12:00 (ZT6) and then decreased gradually until 18:00 (ZT12), when the lights were turned off. During DD, lights were turned off at all times. In both experiments, krill could move freely within a vertical column tank (200 cm height × 50 cm diameter) filled with chilled and filtered seawater (Supplementary Fig. S1). No food was offered at any time during the experiments and an acclimation period of three days (LD, no food) was provided before each experiment. We used an infrared (IR) camera system to monitor DVM during light and dark phases and estimated the variation in mean krill depth over time. We tested the presence of rhythmic patterns using the RAIN algorithm, which allows the detection of rhythms of any period and waveform^23^.

Clear rhythmic oscillations were detected in both experiments. When krill were exposed to LD, they displayed rhythmic vertical migration with a period of about 24 h. Peak upward migration occurred towards the second half of the light phase. In the first experiment (Fig. 1A), RAIN detected a period of oscillation of 24 h (p-value = 3.5e^−07^) in day 1 and a period of 23 h (p-value = 5.4e^−08^) in day 2, with peak upward migration at ZT9 in both days. In the second experiment (Fig. 1B), RAIN detected a period of 24 h (p-value = 4.2e^−04^) in day 1, when krill were exposed to LD, with peak upward migration at ZT8.

Krill DVM in the field displays mostly a nocturnal migratory pattern, characterized by upward migration during the night and downward migration during the day^6,11^. Therefore, we were at first surprised to observe the opposite DVM pattern in the lab, with upward migration during the day (i.e. light phase) and downward migration during the night (i.e. dark phase). However, previous studies on *Drosophila* already revealed that circadian behaviors might differ significantly between wild and laboratory populations of the same species^24^. Physical constriction, exposure to artificial light regimes and feeding schedules and deprivation of ecological interactions may have a profound impact on the behavior of captured animals^25^. In the aquarium, krill are fed mostly during the day. This, in association with the absence of predators, might have led to the reversal of the DVM rhythm. In addition, in order to avoid interaction with rhythmic cues related to food, krill were briefly starved before and during the experiment. Previous studies suggested that starved zooplankton might become more attracted towards light sources^26,27^. This may have contributed to stimulate the upward movement observed during the light phases. Additional observations of DVM with (A) krill accustomed to different feeding schedules (e.g. krill fed during the night) and (B) starved krill exposed to illuminated dark phases (DL or LL) might help to clarify these points.

After we switched to DD conditions (Fig. 1B), during the first day we did not observe any significant rhythm. This might not necessarily imply that no rhythmic migration occurred. In fact, individual krill might have been still rhythmic, but they might have not been synchronized with each other due to the absence of the LD entraining cue or *Zeitgeber*. A similar phenomenon has already been reported for wild zooplankton in the Arctic and Antarctic during periods of continuous illumination in summer (i.e. during midnight sun)^28,29^. At that time of the year, in the Arctic, the local populations of *Calanus finmarchicus* and *C. glacialis* did not display any net DVM as revealed by backscatter data, but unsynchronized individual migrations were registered instead^29^. Implementation of individual tracking methods to apply in the lab during DVM monitoring under extreme photoperiodic conditions including constant darkness (DD) and constant light (LL) would allow further insight into this aspect.

During the second day of DD exposure, RAIN detected a period of 12 h (p-value = 2.3e^−02^), with a first peak of migration in the early subjective light phase (CT29) and a second peak in the early dark phase (CT41). This suggested that in free-running conditions an endogenous rhythm of vertical migration might emerge with a period of about 12 h. This is in agreement with previous studies of swimming activity, oxygen consumption, enzyme activity and transcription in krill exposed to DD^16,18,22^ and strongly suggests that the endogenous clock in krill is characterized by a free-running period significantly shorter than 24 h.

### Oxygen consumption

An endogenous rhythm of activity occurring at the organismic level might contribute to the occurrence of DVM in zooplankton^19,30^. This might apply to krill as well, since they are known to display daily rhythms in metabolism and physiology^15,16^. Therefore, we monitored daily rhythms of oxygen consumption in krill exposed to LD and DD and associated them with the observed DVM patterns. Due to technical limitations, we could not monitor oxygen consumption directly within the vertical migration tank. We used individual krill incubated in 2 L Schott bottles filled with oxygen-saturated chilled and filtered seawater instead. We followed the decrease in *pO*_2_ over a period of 48 h and tested the presence of rhythmic consumption patterns using the RAIN algorithm. Clear rhythmic oscillations were detected in both conditions.

**Figure 2:**
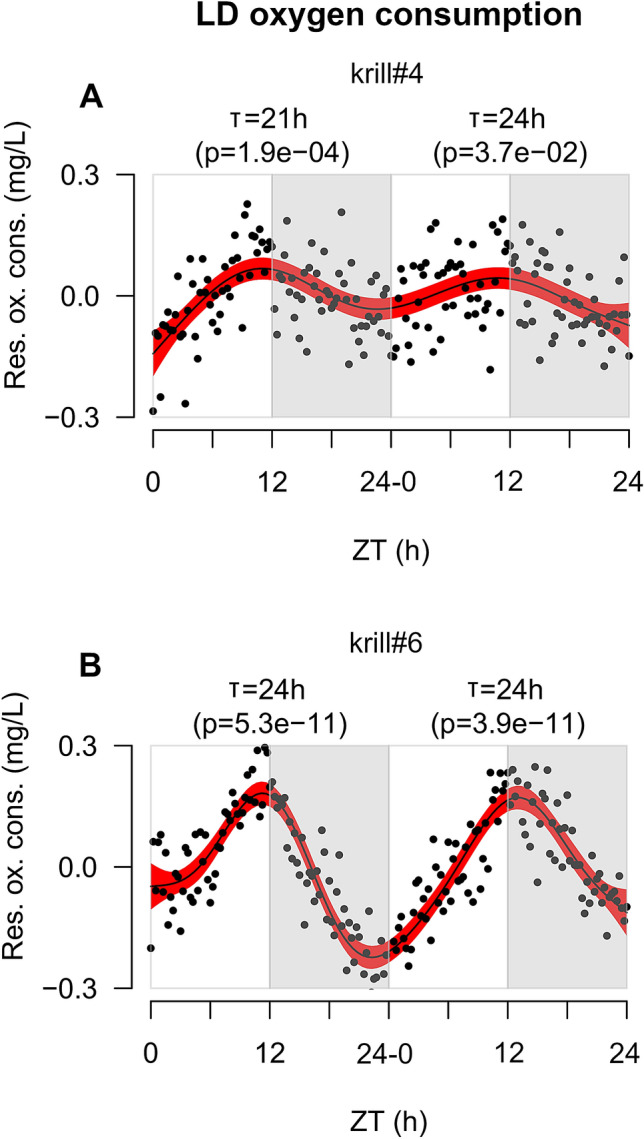
Oxygen consumption patterns of krill exposed to LD. (**A**,**B**) In both panels, X-axis represents time given as Zeitgeber Time or ZT measured in hours. ZT0 corresponds to each event of lights-on (6:00). Y-axis represents residual oxygen consumption expressed in mg/L. Positive values indicate increase of oxygen consumption, whereas negative values indicate decrease of oxygen consumption. Black points represent raw data points. Black solid line represents the model fit obtained by applying a GAM to the residual oxygen consumption over time. Red-shaded areas represent the 95% confidence interval around the GAM model’s fit. White and grey rectangles represent alternation of light and dark phases. For each experimental day, results of the RAIN analysis test for the presence of rhythmic oscillations are reported (T = period of oscillation; p = p-value). The figure was generated using the *plot* function in the “graphics” package (version 3.6.3, https://www.rdocumentation.org/packages/graphics) in R (RStudio version 1.0.136, RStudio Team 2016).

In LD, two out of six tested animals (33%) displayed rhythmic oxygen consumption (Fig. 2A,B). Both individuals displayed clear oscillations in the circadian range (i.e., approx. 24 h period): the first one (krill#4, Fig. 2A) displayed a period of 21 h in day 1 (p-value = 1.9e^−04^) and 24 h in day 2 (p-value = 3.7e^−02^), while the second one (krill#6, Fig. 2B) displayed a period of 24 h in both days (day 1, p-value = 5.3e^−11^; day 2, p-value = 3.9e^−11^). The oscillation was always strongly synchronized to the LD cycle, with peak oxygen consumption occurring at ZT11 (day 1) and ZT12 (day 2) in krill#4 (Fig. 2A) and ZT12 (day 1) and ZT13 (day 2) in krill#6 (Fig. 2B), corresponding to the light/dark transitions. Considering that the animals were incubated within a small volume of water (2 L) and their ability of swim freely might have been severely reduced, changes in swimming activity might not have greatly contributed to the oxygen uptake oscillation. Therefore, this could be interpreted as the result of an internal metabolic oscillation instead. This would support the hypothesis that an internal rhythm of activity might contribute to DVM in krill, even if the mechanisms still remain unknown. In the Calanoid copepod *Calanus finmarchicus* similar oscillations of oxygen consumption were interpreted as a metabolic anticipation of DVM^19^.

**Figure 3:**
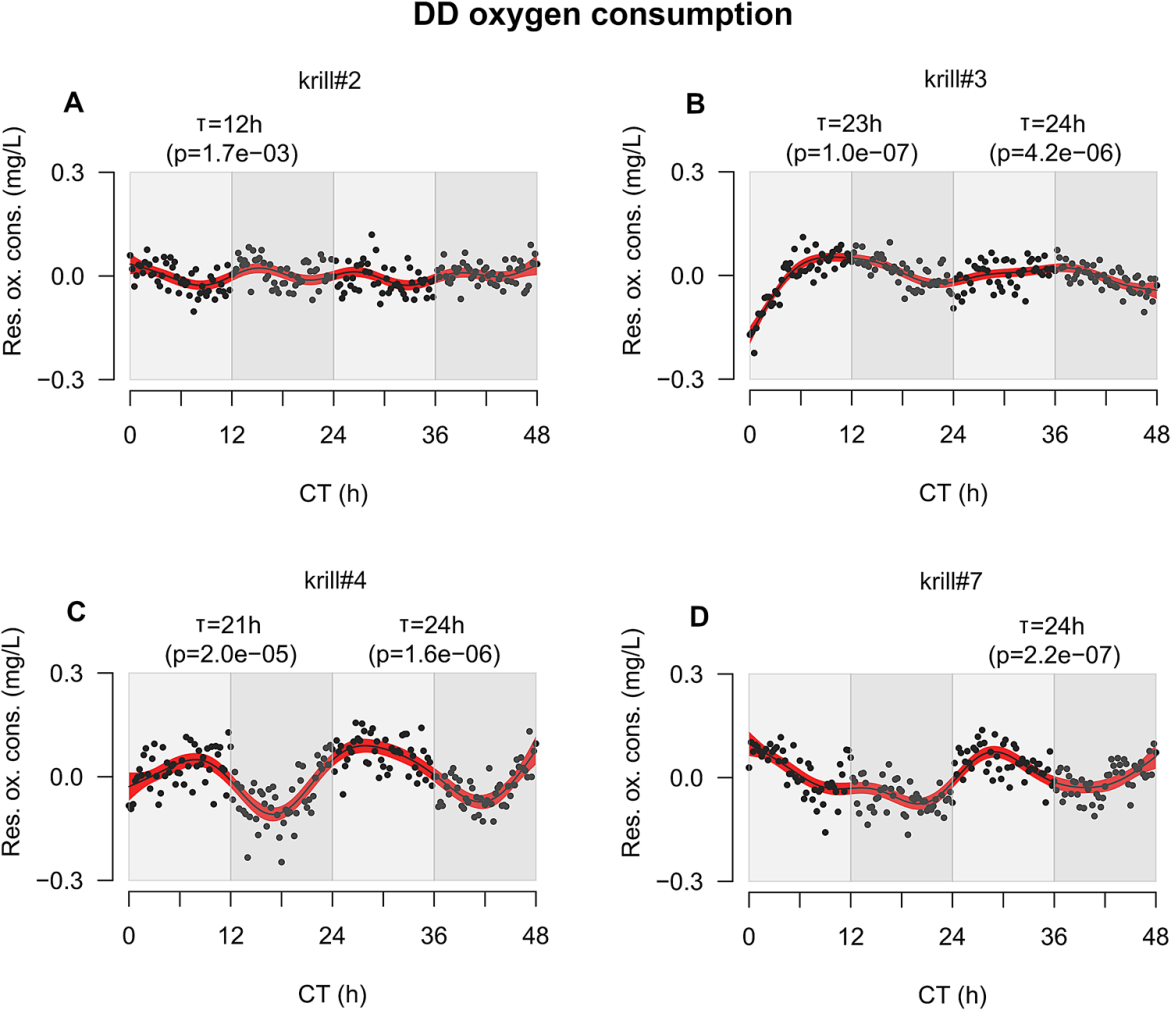
Oxygen consumption patterns of krill exposed to DD. (**A**–**D**) In all panels, X-axis represents time given as Circadian Time or CT measured in hours. CT0 corresponds to first subjective lights-on event (6:00). Y-axis represents residual oxygen consumption expressed in mg/L. Positive values indicate increase of oxygen consumption, whereas negative values indicate decrease of oxygen consumption. Black points represent raw data points. Black solid line represents the model fit obtained by applying a GAM to the residual oxygen consumption over time. Red-shaded areas represent the 95% confidence interval around the GAM model’s fit. Light grey and grey rectangles represent alternation of subjective light and dark phases respectively. For each experimental day, results of the RAIN analysis test for the presence of rhythmic oscillations are reported (T = period of oscillation; p = p-value). The figure was generated using the *plot* function in the “graphics” package (version 3.6.3, https://www.rdocumentation.org/packages/graphics) in R (RStudio version 1.0.136, RStudio Team 2016).

In DD, four out of seven tested animals (57%) displayed rhythmic oxygen consumption (Fig. 3A–D). Three of them (krill#3, #4 and #7, Fig. 3B–D) showed a period of about 24 h. The peak of oxygen uptake was advanced compared to LD and occurred during the subjective light phase. Krill#3 (Fig. 3B) displayed a period of 23 h in day 1 (p-value = 1.0e^−07^) and 24 h in day 2 (p-value = 4.2e^−06^), with peak oxygen consumption at CT11 and CT34. Krill#4 (Fig. 3C) displayed a period of 21 h in day 1 (p-value = 2.0e^−05^) and 24 h in day 2 (p-value = 1.6e^−06^), with peak consumption at CT9 and CT28. Krill#7 (Fig. 3D) displayed a period of 24 h in day 2 (p-value = 2.2e^−07^), with peak consumption at CT28. A fourth individual (krill#2, Fig. 3A) displayed a period of 12 h in day 1 (p-value = 1.7e^−03^), with a first peak of consumption at CT3 during the early subjective light phase and a second peak 12 h later (CT15) during the early dark phase. The general advance of the oxygen uptake peak observed in krill#3, 4 and 7 together with the 12 h rhythm displayed by krill#2 suggested that in free-running DD conditions a process of period shortening similar to the one observed for DVM might occur also for oxygen consumption. Previous studies suggested that in krill similar 12 h free-running endogenous rhythms might occur also at the level of metabolic gene expression and enzymatic activity^16^. This, together with the recent analysis of krill circadian transcriptome revealing a significant 12 h free-running oscillation at the molecular level, strongly suggests that a short endogenous period of oscillation might characterize the circadian clock of Antarctic krill.

The relatively small percentages (33% in LD and 57% in DD) of individual krill showing significant rhythmic oxygen consumption might have been related to the small sample size in our experiment (n = 6 in LD and n = 7 in DD). However, additional measurements of krill oxygen consumption performed using the same methods in LD and DD on larger samples (n = 10), on board of RV *Polarstern* during Antarctic expedition PS 112 (March to May 2018), displayed similar percentages of rhythmic individuals (3 out of 10 or 30% in LD, and 4 out of 10 or 40% in DD) (Supplementary Figs. S2A–C and S3A–D). Interestingly, the on-board analyses revealed the same tendency to develop shorter periods of oscillation in DD (krill#7: T = 12 h, p-value = 8.5e^−04^; krill#9: T = 17 h, p-value = 8.3e^−08^) (Supplementary Fig. S3C,D). Also, on-board measurements in LD displayed almost a reversed phase of oscillation compared to those in the lab, with peak oxygen consumption occurring at the beginning of the light phase, around ZT4 (Supplementary Fig. S2A–C). It is tempting to speculate that this might be related to the reverse DVM pattern observed in the lab. Unfortunately, we do not have the corresponding DVM measurements from the field to support this hypothesis. Additional field observations of krill rhythms of oxygen consumption in association with DVM would help to clarify how these two phenomena might be related to each other.

### Dissection of putative circadian oscillators in krill brain and eyestalks

It might be possible that the DVM rhythm and the metabolic oscillation observed in krill were under the influence of separated circadian oscillators. This was suggested by the different responses displayed by DVM and oxygen consumption in DD. While DVM showed a complete shift to a 12 h rhythm, oxygen consumption only showed an advance in the peaking time.

Previous studies on the circadian system of Crustaceans identified multiple oscillators located in the head and along the body^31^. In particular, a model has been proposed where the circadian oscillators in the head are situated in the brain, in the eyestalks and in the retinae of the compound eye (Supplementary Fig. S4)^31^. To investigate this hypothesis we compared, for the first time, daily patterns of clock genes expression in brain and eyestalks tissue of krill exposed to LD and DD. Krill were sampled within a 72-h time-series, 9 animals were collected every four hours. During the first 24 h (ZT0-24), krill were exposed to LD, while during the remaining 48 h (CT0-48) krill were exposed to DD. We dissected brain and eyestalks tissue (Supplementary Fig. S5) and measured relative changes in clock-related mRNAs over time during the first day in LD (ZT0-24) and during the second day in DD (CT24-48) (Supplementary Table S1). We used RAIN to check for rhythmic oscillations within tissue.

**Figure 4:**
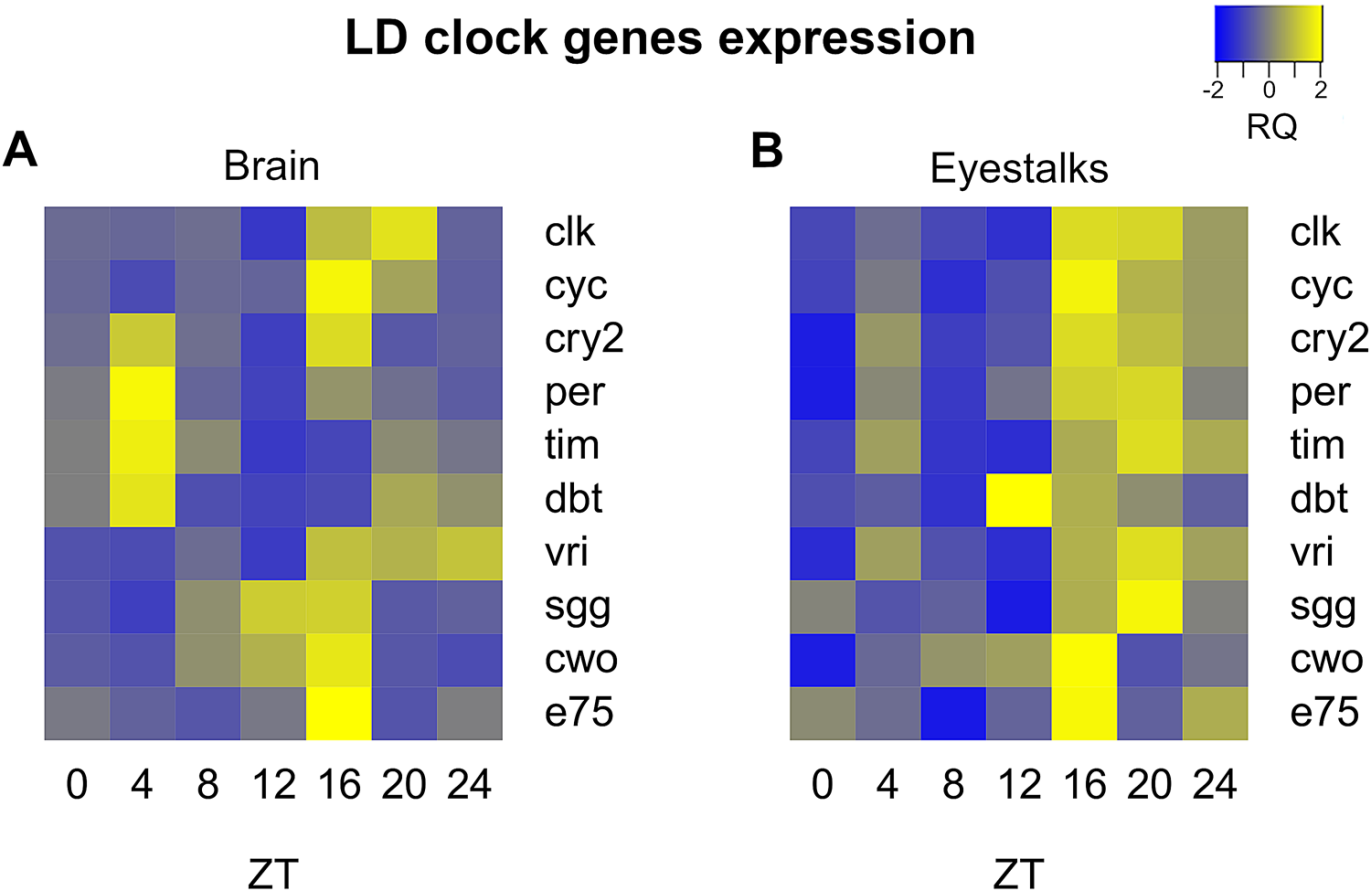
Comparison of daily patterns of clock genes expression in LD between brain and eyestalk tissues. (**A**, **B**) Heatmaps representing up- (in yellow) and down- (in blue) regulation of clock genes expression over time in the brain (**A**) and eyestalks (**B**) of krill exposed to LD. For gene names abbreviations please see Supplementary Table S1. Zeitgeber Time (ZT) given in hour from time of lights-on (6:00), corresponding to ZT0, is indicated below each column of the heatmaps. Heatmaps were generated using the *heatmap.2* function in the “gplots” package (version 3.0.4, https://github.com/talgalili/gplots) in R (RStudio version 1.0.136, RStudio Team 2016).

Clear rhythms of oscillation were found in both tissues. In LD, the core clock components in the brain displayed an antiphase relationship between positive (clk-cyc) and negative (per-tim) regulators, with clk-cyc peaking around ZT16-20 (clk: T = 24 h, p-value = 4.86e^−07^; cyc: T = 24 h, p-value = 2.28e^−12^) and per-tim peaking around ZT4 (per: T = 24 h, p-value = 2.99e^−05^; tim: T = 24 h, p-value = 1.31e^−11^) (Fig. 4A). In the eyestalks such antiphase relationship was not present and most clock genes showed upregulation during the dark phase (ZT16-24) (Fig. 4B). The antiphase relationship is a typical feature of the circadian clock and is well-known from studies on model organisms like *Drosophila* and mouse^2^. So far, previous studies on krill failed to demonstrate a clear antiphase relationship between positive and negative regulators^21,22^. This was discussed as a non-canonical feature of krill circadian clock, as observed before in other Crustacean species^32,33^. However, our results suggest that this might be related to the specific tissue analyzed. In fact, previous works focused mostly on the eyestalks or on the full head^21,22^, possibly failing to detect the antiphase oscillation in the brain. This indicates that separate oscillators might be present in the brain and the eyestalks of krill, as already suggested for other Crustaceans^31^, and suggests that the central pacemaker might be located in the brain. The oscillator in the eyestalks, where all clock genes displayed upregulation during the dark phase, seems to be mostly influenced by the external photoperiod and might be considered as a peripheral oscillator.

**Figure 5:**
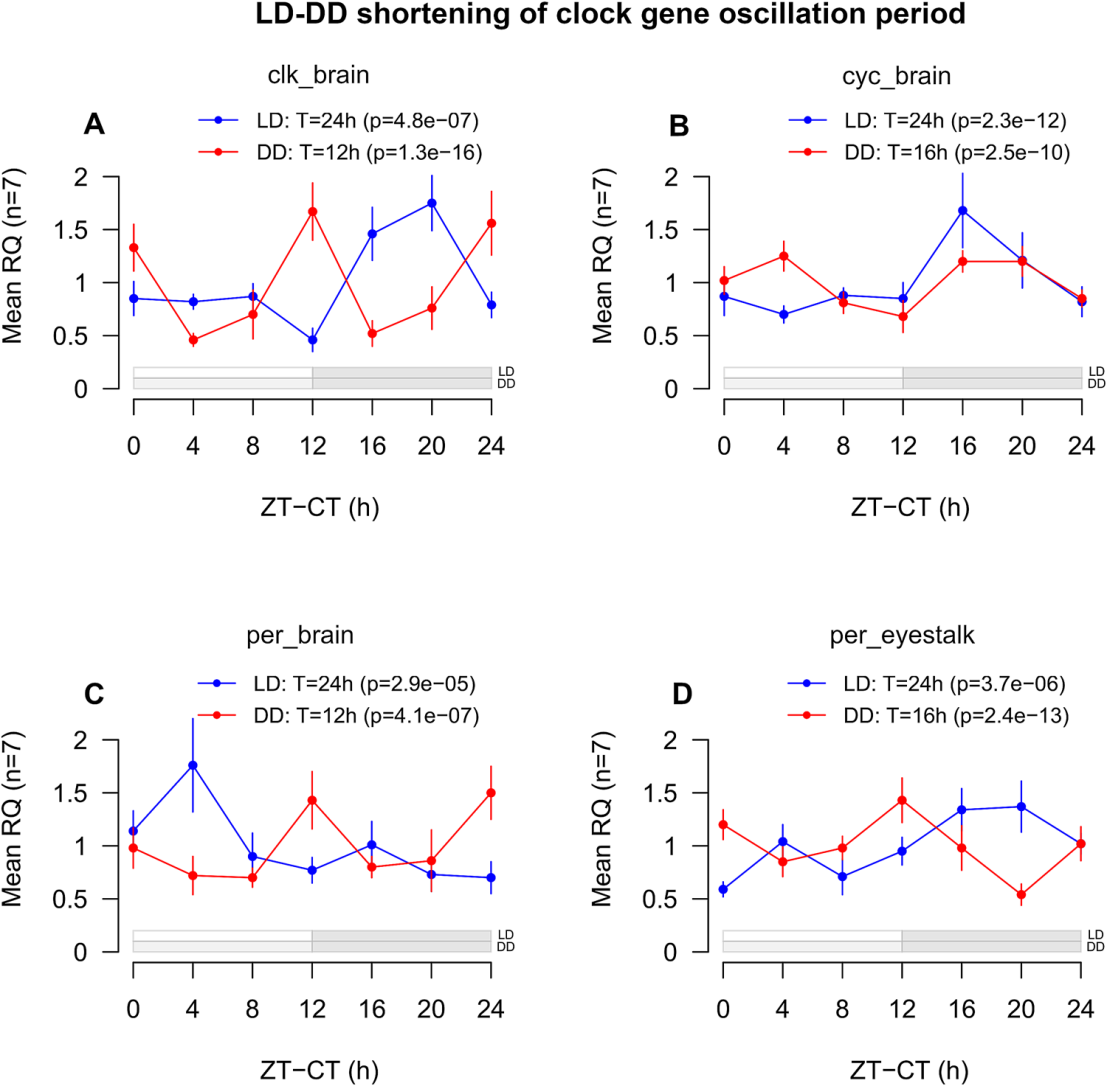
LD-DD shortening of the oscillation period in the core clock genes clk, cyc and per. (**A–C**) Line-plots representing changes in expression levels over time for the clock genes clk (**A**), cyc (**B**) and per (**C**) in LD (blue) and DD (red) in the brain, and for the clock gene per (**D**) in LD (blue) and DD (red) in the eyestalks. Time intervals are reported on the x-axis. For LD, Zeitgeber Time (ZT) is used, given in hours from the time of lights-on (6:00). For DD, Circadian Time (CT) is used, given in hours from the beginning of the subjective light phase (6:00). Mean gene expression levels (n = 7) are reported on the y-axis as mean Relative Quantities (RQ), indicating the average normalized expression levels of the target clock genes relative to the expression levels of the selected internal and external reference genes at each time interval. Error bars represents SEMs (n = 7). Results of the RAIN analysis test for the presence of rhythmic oscillations are reported (T = period of oscillation; p = p-value). A schematic representation of the light/dark cycle is given below the graphs. For LD, withe rectangles indicate light phases, grey rectangles indicate dark phases. For DD, light grey rectangles indicate subjective light phases, grey rectangles indicate dark phases. The figure was generated using the *plot* function in the “graphics” package (version 3.6.3, https://www.rdocumentation.org/packages/graphics) in R (RStudio version 1.0.136, RStudio Team 2016).

As in LD, also in DD different patterns of regulations were observed in the brain and in the eyestalks (Supplementary Fig. S6A,B). In the brain, clk, cyc and per, three of the core clock components, displayed a marked tendency towards a shortening of the oscillation period (Fig. 5A–C). Clk shifted from 24 to 12 h (p-value = 1.31e^−16^), cyc from 24 to 16 h (p-value = 2.48e^−10^) and per from 24 to 12 h (p-value = 4.05e^−07^). In the eyestalks, only per showed a similar tendency, shifting from 24 h (p-value = 3.69e^−06^) to 16 h (p-value = 2.36e^−13^) (Fig. 5D). This again suggested that separated oscillators might be present in krill brain and eyestalks. The shortening of clock gene oscillation period observed in DD in the brain and, to a lesser extent, in the eyestalks, together with the short (12–16 h) oscillation period registered for cry2 across all tissues and conditions (brain LD: T = 12 h, p-value = 2.4e^−08^; brain DD: T = 12 h, p-value = 1.4e^−04^; eyestalk LD: T = 12 h, p-value = 3.2e^−05^; eyestalk DD: T = 16 h, p-value = 6.1e^−13^) (Supplementary Fig. S7A,B), is in agreement with previous observations of krill clock gene expression in DD^22^ and strongly indicates that krill endogenous circadian period might be significantly shorter than 24 h also at the molecular level.

The presence of separated oscillators in krill might help to coordinate different aspects of daily rhythmicity on different levels including physiology (e.g. oxygen consumption) and behavior (e.g. DVM). This might allow a flexible regulation of daily rhythms in response to local changes in environmental conditions driven by temporary and/or seasonal factors including photoperiod, food availability and presence/absence of predators among others.

### Adaptive significance of the short endogenous free-running period in krill

The tendency in krill to display a 12 h endogenous free-running period might represent a circadian adaptation for living at high latitudes, where the photoperiodic signal displays strong seasonal variability^16,22^. Surveys from plants and insects along broad latitudinal gradients indicate that high-latitude species tend to have shorter endogenous periods compared to low-latitude ones^34^. The reasons why this happens are not fully understood yet, but it has been proposed that the short endogenous period might help the clock to entrain to a wider range of photoperiods^34^. Indeed, krill displayed rhythmic clock gene activity during the midnight sun in summer, suggesting that their clock can successfully entrain to extremely long photoperiods^17,35^. However, exposition to similar extreme photoperiods in the laboratory apparently caused the disruption of the clock, suggesting that in the field krill might switch to alternative *Zeitgebers* (i.e. entraining cues) to entrain the clock when photoperiod becomes extremely long/short (e.g. midsummer/midwinter)^36^.

From an ecological perspective, having a short endogenous period might help krill to adapt to different environmental scenarios. In the presence of overt day/night cycles, the clock would entrain to the photoperiod and promote daily rhythms with a period of approx. 24 h, like for example the nocturnal DVM pattern usually observed during spring and autumn. This would help krill to anticipate the day/night cycle and maximize the costs/benefits balance between time spent feeding at the surface and time spent hiding in deeper water layers. At the same time, the clock would promote 24 h oscillations in krill metabolism and physiology and coordinate phases of high and low activity with phases of upward and downward migration during DVM.

On the other hand, in the absence of overt day/night cues, for example during summer and winter, the clock would tend to shift towards the free-running period and promote daily rhythms with a period shorter than 24 h. Observations of krill DVM in the field during winter are scarce, due to the harsh weather conditions which characterize the Southern Ocean at that time of the year. Much of the rhythmic biology of krill during winter still remains unknown. At the same time, field observations of krill DVM and swimming activity during summer suggest that a de-synchronized, “around-the -clock”, individual migratory movement might be present instead^18,28^. According to some studies, frequent shallow individual migrations could occur during summer as a result of a hunger-satiation mechanism, triggered by the abundant primary production occurring in the surface layers^37^. To get further insight into these aspects, first we would need to perform additional laboratory studies and field observations of krill rhythmic functions (oxygen consumption, swimming activity, DVM) under extreme photoperiods (LL-DD in the lab, summer–winter in the field). Second, laboratory and on-board trials with krill exposed to different food concentrations (low-medium–high) and different photoperiods (LD-DD-LL) could be used to study the interaction between food and light cues in the emergence of “opportunistic” rhythmic responses.

## Conclusion

Our results indicate that in krill important putative clock-output functions like DVM and oxygen consumption display a similar shortened free-running period of oscillation as previously described for the clock itself and for the transcriptome. In addition, we collected evidence that in krill separated circadian oscillators might occur in the brain and in the eyestalks, and a shortening of the oscillation period was observed in DD in the brain. Since daily rhythms in physiology and behavior have a great impact on krill survival, especially those related to DVM, our results support the hypothesis that the short free-running period of the clock might represent a circadian adaption for krill living at high-latitudes.

## Methods

### Ethics statement

All animal work was conducted according to relevant national and international guidelines. Krill catches, welfare and experimentation were based on permission from the Department of Environment and Heritage of the Australian Government and were conducted in accordance with the Antarctic Marine Living Resources Conservation Act 1981 (permit number: 06_09_2220) and the Environment Protection and Biodiversity Conservation Act 1999 (permit number: WT2007-1480).

### Animals

Krill were caught in East Antarctica (66° 47′ S, 65° 08′ E) on Feb 12th 2013 at 17:43 UTC in the upper 30 m of the water column using a rectangular midwater trawl (RMT 8) during voyage V3 12/13 of RSV *Aurora Australis*. On board, krill were kept in 200 L tanks, at constant temperature (0 °C), under dim light, with no food and provided with a continuous supply of chilled seawater. After arrival in Hobart, Tasmania, on Feb 22th 2013, krill were transported to the research aquarium at the Australian Antarctic Division (AAD) in Kingston, where they were transferred into 1670 L tanks connected to an 8000 L seawater recirculation system. Conditions of maintenance of krill in the aquarium including illumination and feeding have been described in detail elsewhere^38,39^.

### Vertical migration

To monitor vertical migration, we used small groups of krill which were free to move within a vertical column tank (Supplementary Fig. S1). We performed two experimental runs. In the first run, we monitored DVM of a group of 45 krill exposed to LD for 48 h. Lights were turned on at 6:00 (ZT0). Light intensity gradually increased until 12:00 (ZT6) reaching the maximum of 50 lx at the surface of the tank, then decreased until 18:00 (ZT12), when lights were turned off until 6:00 the next morning (ZT24). Light was provided by one fluorescent tube placed above the tank at 50 cm distance covered with a gel filter simulating light attenuation at 30 m depth in natural conditions (ARRI, Marine Blue 131). This is the same method used to illuminate the holding tanks of the aquarium and no apparent vertical light intensity gradient was detected within and around the column, which resulted illuminated in a homogenous way. In the second run, we used a group of 41 krill exposed to LD for 24 h followed by constant darkness (DD) for 48 h. For both runs, adult krill of mixed sexes and 30–35 mm length were used. The experimental tank was a vertical cylinder of transparent acrylic (200 cm × 50 cm) connected to the water circulation system of the aquarium. Before each run, an acclimation period of three days was provided, with LD and no food. No food was offered during the runs and no animals died during the experiments. Vertical migration was monitored continuously during light and dark phases using an infrared-sensitive camera system (SJ4000, SJCAM) together with an infrared (λ = 850 nm) illumination system (Camera 2000 Limited). Every 30 min, a snapshot was taken and by manually examining the snapshots two independent observers estimated changes in krill mean depth over time. We used the RAIN algorithm to test for rhythmic oscillations having a period included between 12 and 24 h^23^. Each experimental day was tested separately from the others.

### Oxygen consumption

Oxygen consumption was monitored using single krill incubated individually in 2 L Schott bottles filled with oxygen-saturated chilled and filtered seawater. We performed two runs, one in LD (same LD cycle used for DVM) and one in DD. Both runs lasted 48 h. During each run, 7 krill (mixed sexes; 30–35 mm length) were monitored in parallel using a 10-channel fiber optic oxygen transmitter (Oxy-10 Mini, PreSens) in combination with type PSt3 sensors and the Oxy-10 software (PreSens). In LD, one channel had a failure giving the final sample sizes of n = 6 in LD and n = 7 in DD. Three bottles with no krill served as controls during each run. Krill were starved for 6 h prior incubation, to avoid interaction with digestive processes. During the runs, the bottles were placed inside the holding tank to ensure constant temperature (0.5 °C). Oxygen saturation was measured every 15 min and normalized against the controls’ average. We de-trended the data applying a linear model using the *lm* function in R (RStudio version 1.0.136, RStudio Team 2016) and extracted the residuals using the *residuals* function. To check the presence of a temporal pattern in the distribution of the residuals we applied a generalized linear model (GAM) using the *gam* function in the “mgcv” R package^40^. We finally used RAIN to test for rhythmic oscillations in the residuals having a period included between 12 and 24 h. We tested each day separately.

### Gene expression

To examine daily patterns of clock genes expression in krill brain and eyestalks, we used adult krill of mixed sexes (30–35 mm length) exposed to LD (same as DVM and oxygen) and DD. Krill were placed in a separated 200 L tank, connected to the seawater circulation system of the aquarium. One week of acclimation was provided with LD and low food. Feeding was interrupted 72 h before the beginning of the sampling. The time-series sampling lasted 72 h and 9 animals were randomly collected every 4 h. During the first 24 h (ZT0-24) krill were exposed to LD, while during the remaining 48 h (CT0-48) they were exposed to DD. No feeding occurred during the sampling. Sampling in the darkness was conducted using dim red light.

For each collected animal, we immediately cut the head following an oblique line just behind the eyes, to avoid contamination with stomach tissue, and incubated it in RNAlater at 4 °C for 24 h. After that, we dissected brain and eyestalks (Supplementary Fig. S5) and placed them separately in fresh RNAlater stored at − 80 °C. We extracted total RNA using the Direct-zol RNA MicroPrep kit (Zymo Research, USA) with a genomic DNA digestion step included. We checked for RNA purity using a Nanodrop 2000 Spectrophotometer (ThermoScientific) and for RNA integrity using an Agilent 2100 Bioanalyzer system (Agilent Technologies). We measured gene expression of 10 clock genes (Supplementary Table S1) using SYBR Green chemistry (GoTaq 1-Step qPCR System, Promega) on a CFX384 Touch Real-Time PCR Detection System (Bio-Rad). 10 ng of total RNA was PCR amplified in 10 μL total volume. Primers were designed around the sequences of interest published in^21^ or available from Krilldb online database^20^. Primer efficiencies and specificity of the amplicons were assessed by standard curves and dissociation curves, respectively.

To normalize the qPCR data, we used a combination of internal and external controls. As internal control we used the housekeeper gene usp46, which was used before to normalize clock genes expression data in krill exposed to similar LD conditions^36,41^. As external control we used an exogenous RNA “spike-in” control, which had been used before for the determination of clock gene expression levels in krill caught during summer in Antarctica^21^. The spike was synthesized from 1.5 µg of DNA template using the MAXIscript T3 Transcription Kit (Thermo Fisher Scientific). The purified spike was added to each RNA sample at a constant concentration (10 pg). The primers for usp46 and for the spike were designed around the sequence of interest available from the Krilldb online database^20^ and GenBank respectively (Supplementary Table S1). Raw Cq (quantification cycle) values were normalized using the modified 2^−ΔΔCt^ method, which takes into account gene-specific amplification efficiencies and allows for combination of multiple reference genes^42^. In order to reduce the high variability typically observed in independent time-series, the lower and higher RQ values of each time-point were discarded. Normalized relative quantities (NRQs) were calculated on the average RQ value among all time-points. We used RAIN to test for rhythmic oscillations having a period between 12 and 24 h in the temporal variation of mean NRQs (n = 7) of the target clock genes over the 24 h cycle^23^.

## Supplementary information

Supplementary Information.
