# Supplementary material for: Circadian regulation of diel vertical migration (DVM) and metabolism in Antarctic krill Euphausia superba
Source: Sci Rep. 2020 Oct 8;10:16796. doi: 10.1038/s41598-020-73823-5 (PMC7546626; doi:10.1038/s41598-020-73823-5)
Supplement: Supplementary file 1 — Supplementary Information. [file 41598_2020_73823_MOESM1_ESM.docx]

**Circadian regulation of diel vertical migration (DVM) and metabolism in Antarctic krill *Euphausia superba*.**

Fabio Piccolin^1*^, Lisa Pitzschler^1^, Alberto Biscontin^2^, So Kawaguchi^3^, Bettina Meyer^1,4,5*^.

^1^Alfred Wegener Institute Helmholtz Centre for Polar and Marine Research, Am Handelshafen 12, 27570 Bremerhaven, Germany

^2^University of Padova, Department of Biology, via Ugo Bassi 58/b, 35121 Padova, Italy.

^3^Australian Antarctic Division, Department of the Environment and Energy, 203 Channel Hwy, Kingston TAS 7050, Australia.

^4^Institute for Chemistry and Biology of the Marine Environment, University of Oldenburg, Carl-von-Ossietzky-Strasse 9-11, 26111 Oldenburg, Germany.

^5^Helmholtz Institute for Functional Marine Biodiversity at the University of Oldenburg, Ammerländer Heerstrasse 231, 26129 Oldenburg, Germany.

* Correspondence to: fabiopiccolo@hotmail.com; [bettina.meyer@awi.de](mailto:bettina.meyer@awi.de)

**SUPPLEMENTARY FIGURES**


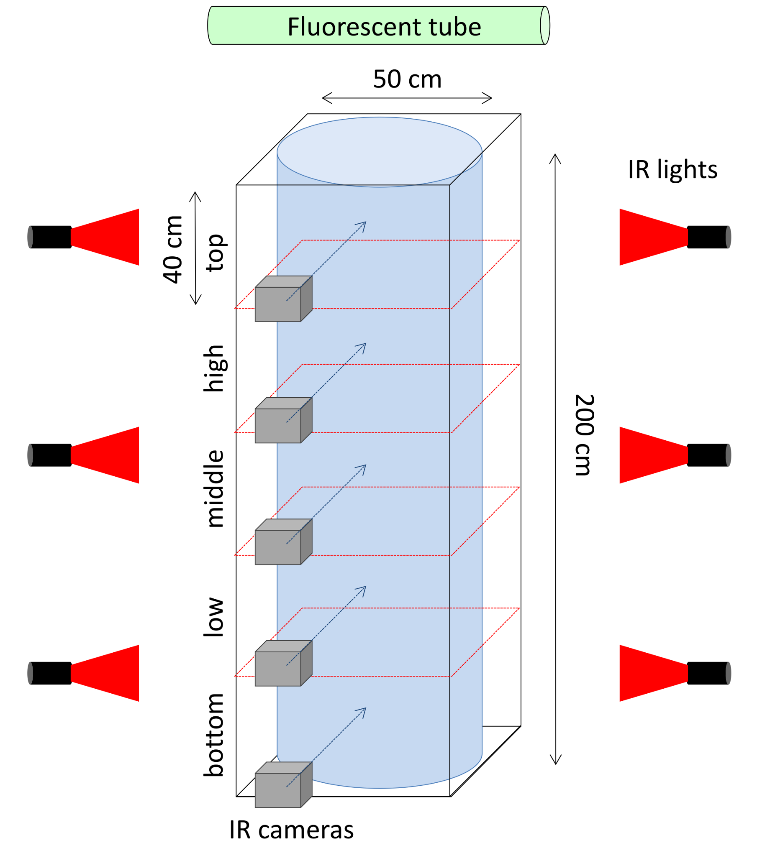


**Supplementary Figure S1. Experimental tank for DVM monitoring.**

The tank used for DVM monitoring was a vertical cylinder of transparent acrylic, 50 cm in diameter x 200 cm in height (light blue-shaded). The tank was filled with filtered and chilled (0.5°C) seawater and connected to the circulation system of the aquarium. The cylinder was placed into a water jacket tank, a column with squared section, also of transparent acrylic, 55 cm in side x 198 cm in height. The water jacket was also filled with filtered and chilled (0.5°C) seawater and connected to the circulation system. Its function was to avoid direct contact of the cylinder with room temperature and keep the temperature within the cylinder constant. Light during light phases was provided by a fluorescent tube placed above the tank, at a distance of approx. 50 cm, shaded with a light-green gel filter simulating light attenuation at 30 m depth within the ocean. The column was conceptually subdivided into 5 height sectors (red lines) and each sector was monitored by a separate IR camera placed in front of the tank at a distance of approx. 40 cm. To illuminate the scene during dark phases, we placed 6 IR illuminators, 3 on each side of the tank perpendicularly to the direction of the cameras (to avoid backlight effects), at increasing height.


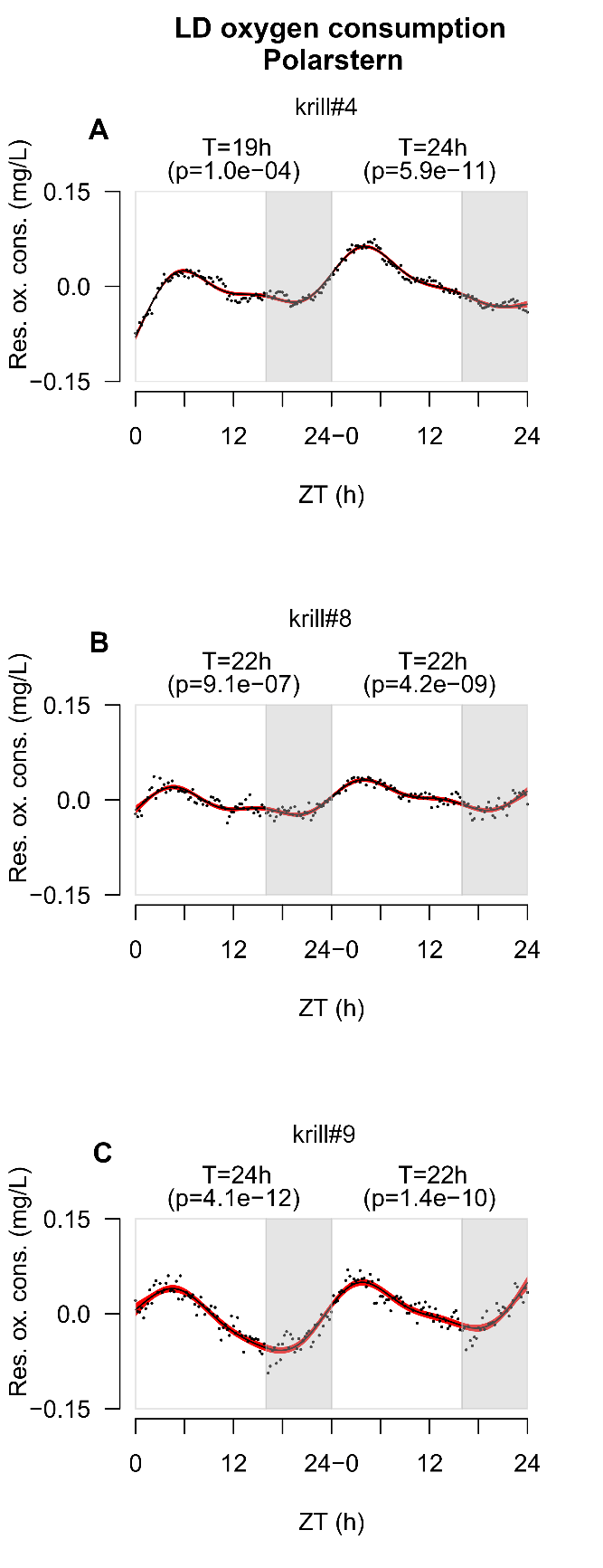


**Supplementary Figure S2. LD oxygen consumption patterns on board of RV *Polarstern*.**

Additional observations of krill oxygen consumption in LD were performed on board of RV *Polarstern* during Antarctic expedition PS 112 (March to May 2018). Krill were caught with a 1.8 m^2^ Isaacs Kidd Midwater Trawl net (IKMT) with a 505 µm mesh and a closed cod end in the upper 20 m of the water column to get life krill in pristine condition. Krill were caught on 8^th^ April 2018 at Elephant Island (Latitude: -61.0, Longitude: -54.8) at 23:55. Sunrise and sunset at fishing location were at 7:30 and 18:00. All times are local time (UTC-4). On board, krill were kept for 24 hours on deck in a flow-through tank with natural seawater of *in situ* temperature, exposed to natural light conditions, until the beginning of the experiments. During the experiment, incubation procedure was the same as described in the paper. The LD cycle used during the experiment was different from the natural light cycle at the fishing station, consisting of 16 h of light (from 7:30 to 23:30) and 8 h of darkness (from 23:30 to 7:30) (LD 16:8). This was done because the krill were already in a state of reduced metabolic activity due to the time of the year (during March-April krill reduce their metabolism to prepare for winter conditions) and we had to stimulate them with longer photoperiods. Oxygen consumption was monitored using 3 x 4-channels fiber optic oxygen transmitters (Oxy-4 Mini, PreSens) in combination with type PSt3 sensors and the Oxy-4 software (PreSens). The 3 transmitters were used in parallel, giving a total number of 12 channels. During the experiment, 10 channels were connected to bottles with krill and 2 channels were connected to bottles without krill and used as controls. Data collection and analysis were implemented as described in the paper.

**A-C.** In all panels, X-axis represents time given as Zeitgeber Time or ZT measured in hours. ZT0 corresponds to each event of lights-on (7:30). Y-axis represents residual oxygen consumption expressed in mg/L. Positive values indicate increase of oxygen consumption, whereas negative values indicate decrease of oxygen consumption. Black points represent raw data points. Black solid line represents the model fit obtained by applying a GAM to the residual oxygen consumption over time. Red-shaded areas represent the 95% confidence interval around the GAM model’s fit. White and grey rectangles represent alternation of light and dark phases. For each experimental day, the results of the RAIN test are reported (T = period of oscillation; p = p-value). The figure was generated using the *plot* function in the “graphics” package (version 3.6.3, https://www.rdocumentation.org/packages/graphics) in R (RStudio version 1.0.136, RStudio Team 2016).


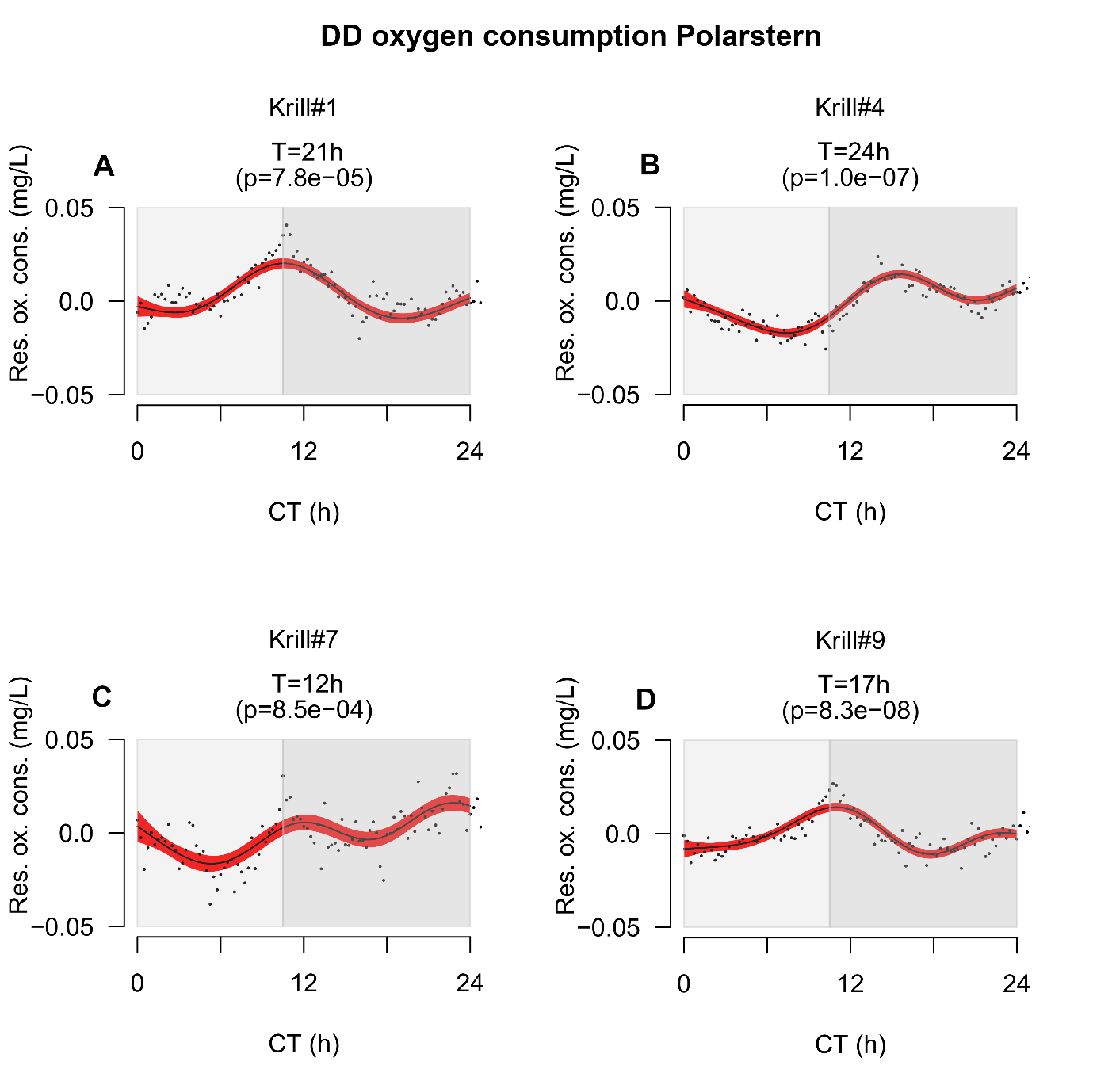


**Supplementary Figure S3. DD oxygen consumption patterns on board of RV *Polarstern*.**

Additional observations of krill oxygen consumption in DD were performed on board of RV *Polarstern* during Antarctic expedition PS 112 (March to May 2018). Krill were caught with a 1.8 m^2^ Isaacs Kidd Midwater Trawl net (IKMT) with a 505 µm mesh and a closed cod end in the upper 20 m of the water column to get life krill in pristine condition. Krill were caught on 13^th^ April 2018 in Bransfield Strait (Latitude: -62.7, Longitude: -54.5) at 18:56. Sunrise and sunset at fishing location were at 7:30 and 18:00. All times are local time (UTC-4). On board, krill were kept for 24 hours on deck in a flow-through tank with natural seawater of *in situ* temperature, exposed to natural light conditions, until the beginning of the experiments. During the experiments, incubation procedure was the same as described in the paper. Oxygen consumption was monitored using 3 x 4-channels fiber optic oxygen transmitters (Oxy-4 Mini, PreSens) in combination with type PSt3 sensors and the Oxy-4 software (PreSens). The 3 transmitters were used in parallel, giving a total number of 12 channels. During the experiment, 10 channels were connected to bottles with krill and 2 channels were connected to bottles without krill and used as controls. Data collection and analysis were implemented as described in the paper.

**A-D:** In all panels, X-axis represents time given as Circadian Time or CT measured in hours. CT0 corresponds to “subjective” lights-on (7:30). Y-axis represents residual oxygen consumption expressed in mg/L. Positive values indicate increase of oxygen consumption, whereas negative values indicate decrease of oxygen consumption. Black points represent raw data points. Black solid line represents the model fit obtained by applying a GAM to the residual oxygen consumption over time. Red-shaded areas represent the 95% confidence interval around the GAM model’s fit. Light grey and grey rectangles represent alternation of subjective light and dark phases. The experiment lasted 24 h, the results of the RAIN test are reported (T = period of oscillation; p = p-value). The figure was generated using the *plot* function in the “graphics” package (version 3.6.3, https://www.rdocumentation.org/packages/graphics) in R (RStudio version 1.0.136, RStudio Team 2016).


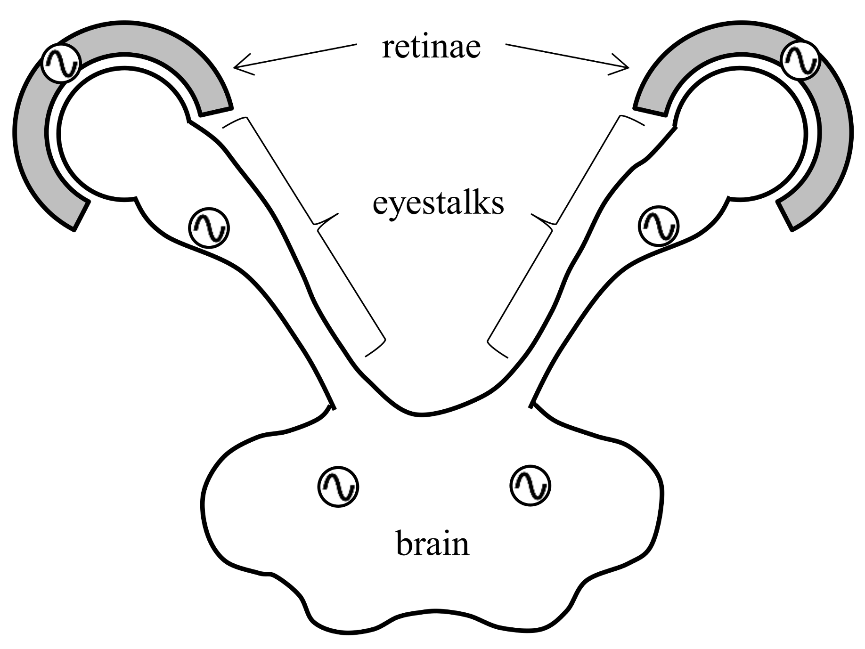


**Supplementary Figure S4. Schematic representation of the circadian system in the head of a generic Crustacean.**

Putative circadian oscillators (represented as small circled sinus waves) in the head of a generic Crustacean are located in the brain, in the eyestalks and in the retinae of the compound eyes. We dissected krill brain and eyestalks and measured clock genes expression within these two tissues. We tried to extract RNA also from the retinae, but we could not obtain sufficiently pure RNA, possibly due to contamination by visual pigments. Figure modified and adapted after Strauss and Dircksen (2010).


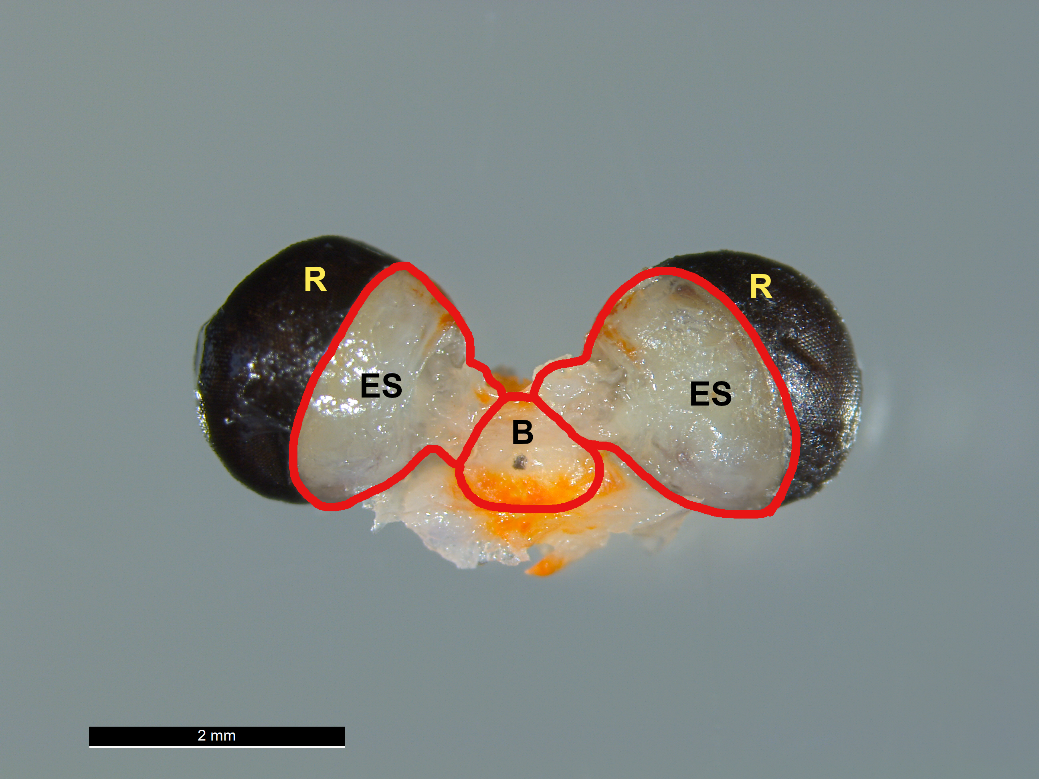


**Supplementary Figure S5. Dissection of krill brain and eyestalk tissues.**

Microscope picture of krill brain (B)-eyestalks (ES)-retinae (R) complex. Red lines indicate the portions of tissue which were used to extract RNA for clock genes expression analysis. Retinae (R) were removed cutting along the edge of the dark-pigmented tissue. Eyestalks (ES) were separated from brain (B) cutting at the root of the stalk. Leftover tissue was removed from the base of the brain (B).


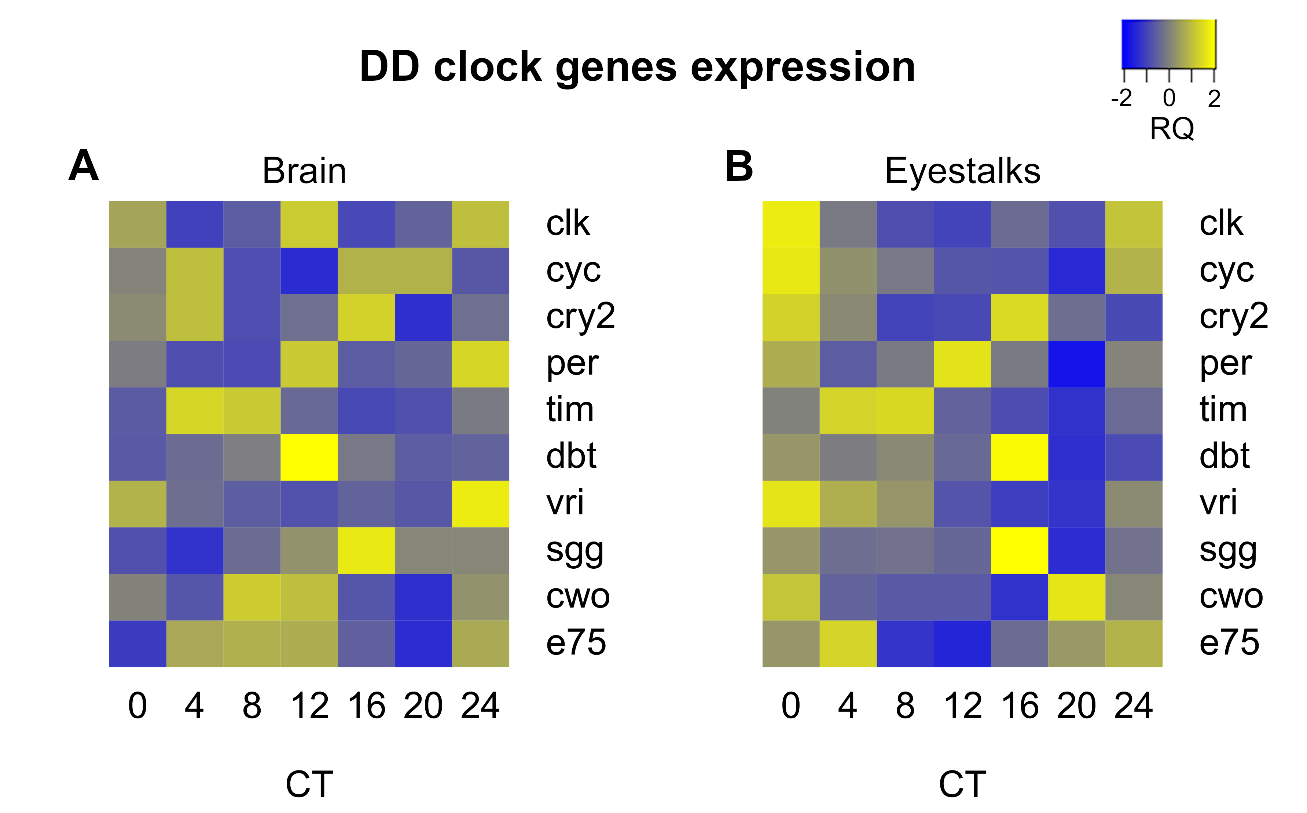


**Supplementary Figure S6. Comparison of daily patterns of clock genes expression in DD between brain and eyestalk tissues.**

**A-B**: Heatmaps representing up- (in yellow) and down- (in blue) regulation of clock genes expression over time in the brain **(A)** and eyestalks **(B)** of krill exposed to DD. For gene names abbreviations please see Supplementary Table S1. Circadian Time (CT) given in hour from time of subjective lights-on (6:00), corresponding to CT0, is indicated below each column of the heatmaps. Heatmaps were generated using the *heatmap.2* function in the “gplots” package (version 3.0.4, https://github.com/talgalili/gplots) in R (RStudio version 1.0.136, RStudio Team 2016).


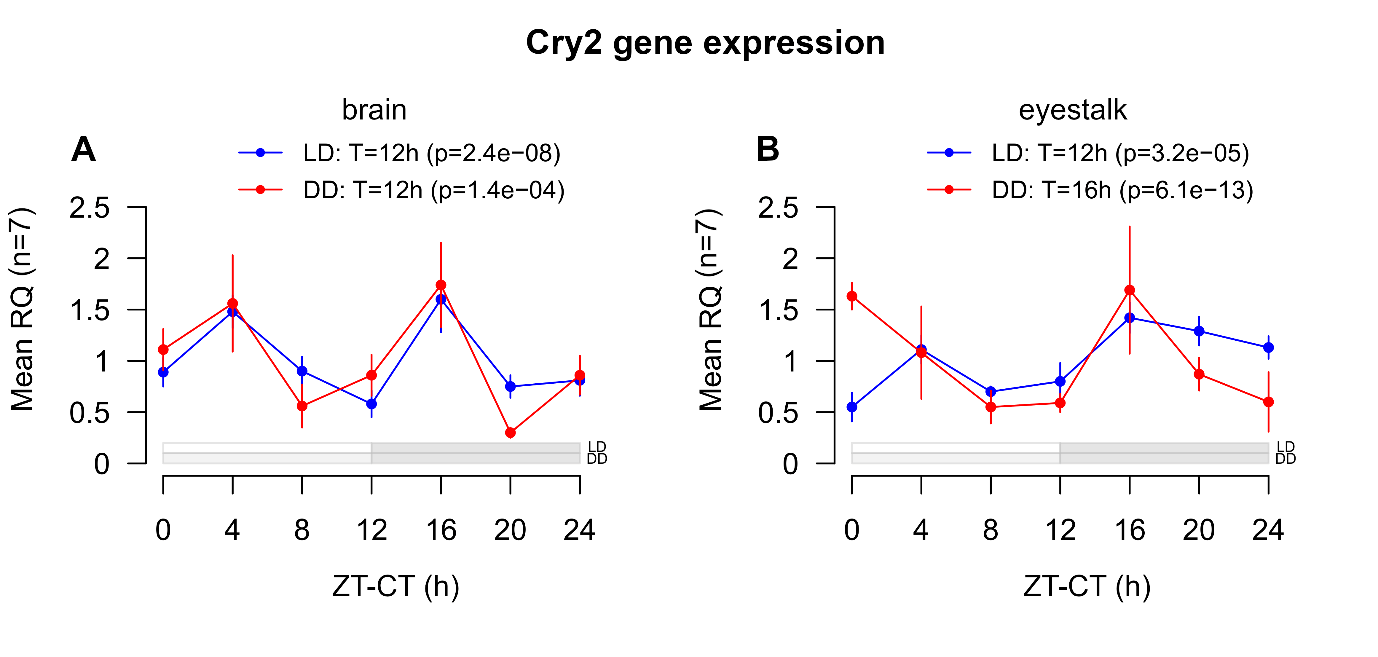


**Supplementary Figure S7. Daily patterns of gene expression of cry2 in LD and DD.**

**A-B**: Line-plots representing changes in expression levels over time for the clock gene cry2 in the brain (**A**) and eyestalks (**B**) of krill exposed to LD (blue) and DD (red). Intervals of Zeitgeber Time (ZT) for LD and Circadian Time (CT) for DD are given in hours on the x-axis. ZT0 corresponds to lights-on (6:00), CT0 corresponds to subjective lights-on (6:00). Mean gene expression levels (n = 7) are reported on the y-axis as mean Relative Quantities (RQ), indicating the average normalized expression levels of the target clock genes relative to the expression levels of the selected internal and external reference genes at each time interval. Error bars represents SEMs (n = 7). Results of the RAIN analysis testing for the presence of rhythmic oscillations are reported (T = period of oscillation; p = p-value). A schematic representation of the light/dark cycle is given. For LD, white rectangles indicate light phases, grey rectangles indicate dark phases. For DD, light grey rectangles indicate subjective light phases, grey rectangles indicate dark phases. The figure was generated using the *plot* function in the “graphics” package (version 3.6.3, https://www.rdocumentation.org/packages/graphics) in R (RStudio version 1.0.136, RStudio Team 2016).

**SUPPLEMENTARY TABLES**


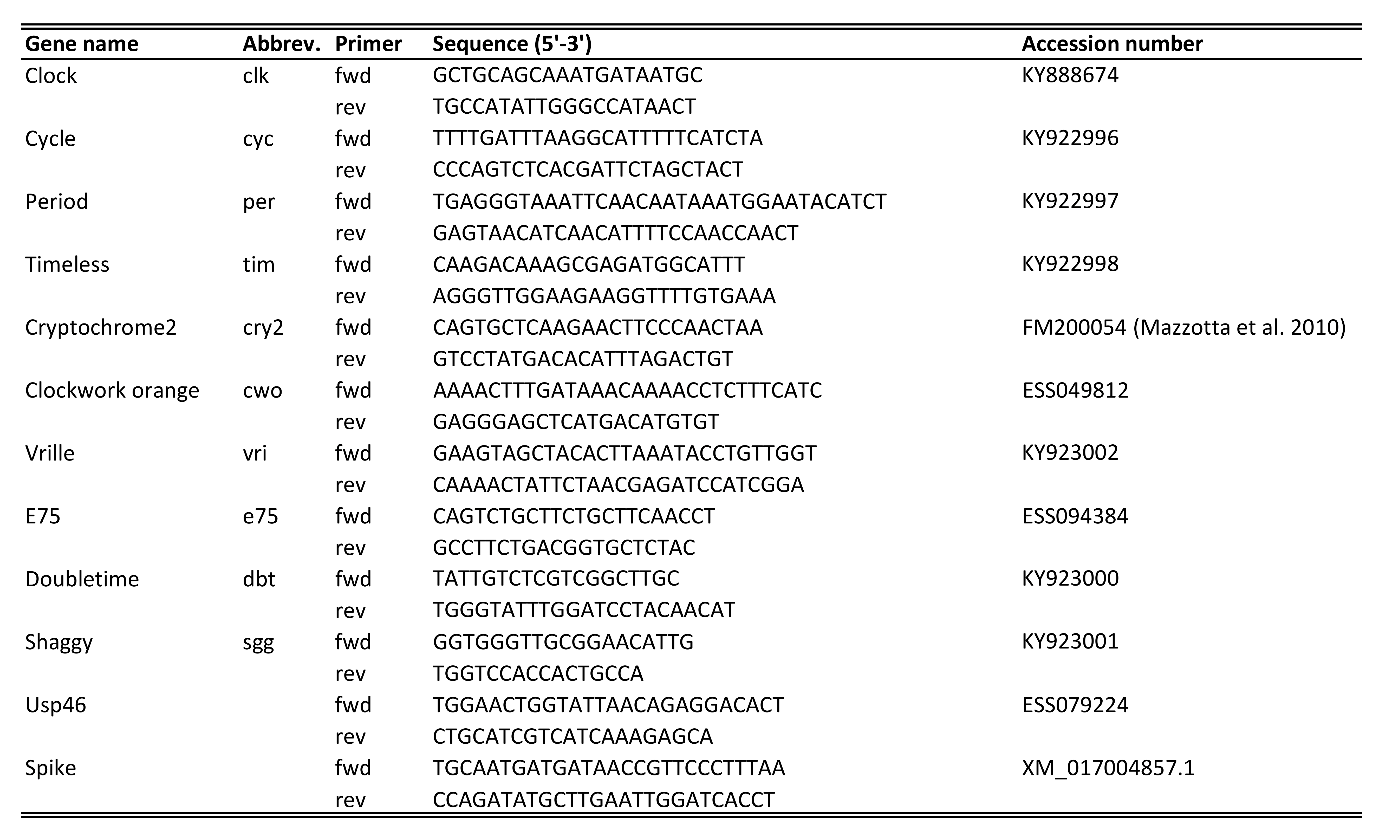


**Supplementary Table S1.**

Primers used for quantitative qRT-PCR to analyze daily profiles of clock genes expression in krill brain and eyestalks tissue in LD and DD. For each gene, the full name is reported, together with the abbreviation used in the text, the forward and reverse primer sequences used for qRT-PCR (direction 5’-3’) and the accession number of the reference sequence used for primer design. Genes denoted as “Usp46” and “Spike” were used respectively as internal and external references for normalization.
